# Estradiol Aggravate *Nocardia farcinica* Infections in Mice

**DOI:** 10.3389/fimmu.2022.858609

**Published:** 2022-03-02

**Authors:** Lichao Han, Xingzhao Ji, Xueping Liu, Shuai Xu, Fang Li, Yanlin Che, Xiaotong Qiu, Lina Sun, Zhenjun Li

**Affiliations:** ^1^ State Key Laboratory of Infectious Disease Prevention and Control, National Institute for Communicable Disease Control and Prevention, Chinese Center for Disease Control and Prevention, Beijing, China; ^2^ Department of Pulmonary and Critical Care Medicine, Shandong Provincial Hospital Affiliated to Shandong First Medical University, Jinan, China; ^3^ Shandong Key Laboratory of Infections Respiratory Disease, Shandong Provincial Hospital Affiliated to Shandong First Medical University, Jinan, China; ^4^ School of Laboratory Medicine and Life Sciences, Wenzhou Medical University, Wenzhou, China; ^5^ Department of Medical, Tibet University, Lhasa, China

**Keywords:** *Nocardia farcinica*, sex difference, 17β-estradiol, estrogen receptor, MAPK

## Abstract

Males are generally more susceptible to *Nocardia* infection than females, with a male-to-female ratio of 2 and higher clinical disease. 17β-Estradiol has been implicated in affecting the sex-based gap by inhibiting the growth of *N. brasiliensis* in experiments, but the underlying mechanisms have not yet been fully clarified. In the present study, however, we report increased severity in *N. farcinica* IFM 10152-infected female mice compared with male mice with increased mortality, elevated lung bacterial loads and an exaggerated pulmonary inflammatory response, which was mimicked in ovariectomized female mice supplemented with E2. Similarly, the overwhelming increase in bacterial loads was also evident in E2-treated host cells, which were associated with downregulating the phosphorylation level of the MAPK pathway by binding the estrogen receptor. We conclude that although there are more clinical cases of *Nocardia* infection in males, estrogen promotes the survival of the bacteria, which leads to aggravated inflammation in females. Our data emphasize the need to include and separately analyze both sexes in future studies of *Nocardia* to understand the sex differences in immune responses and disease pathogenesis.

## Introduction

*Nocardia* is a saprophytic gram-positive bacillus that usually manifests as an opportunistic infection in both immunocompetent and immunocompromised hosts. It is mainly transmitted through the respiratory tract to cause lung abscesses but also through wound or blood transmission to cause skin and central nervous system infections (1, 2).

The genus *Nocardia* currently contains more than 100 species, and clinically, the primary recognized human pathogens include *N. farcinica*, *N. cyriacigeorgica*, *N. brasiliensis* and *N. asteroides*. Nocardiosis has been reported at all ages, and the incidence of males with isolated nocardiosis is significantly higher than that of females worldwide (3, 4), such as in Mexico (5), the United States (6, 7), Canada (8), France (9), Spain (10), Australia (11, 12) and China (13).

However, there is no clear explanation for this sex predominance. One of the most common explanations is that men’s distinct lifestyle- and agriculture-related professions lead to increased exposure to *Nocardia* (14, 15), considering the widespread distribution of this organism, especially in soil, decaying vegetation, fresh water and salt water (1). In addition, the presence of estrogen might also contribute to the sex difference observed (16). As a sex steroid hormone, estrogen exerts a broad spectrum of biological effects by binding to estrogen receptor alpha (ERα) or ERβ (17). Estrogen, primarily 17β-estradiol (E2), regulates cellular function in diverse cell types, including macrophages, dendritic cells (DCs), granulocytes, and lymphocytes. It is important to mention here that E2 has divergent effects on inflammation controls. It diminishes the severity of infections by some pathogens, whereas it enhances susceptibility to other pathogens (18, 19). This aroused our interest in further investigating the role and mechanism of E2 in *Nocardia* infection.

Given the sex hormone and genetic and physiological differences between the sexes, males and females differ in their immune responses to infection with many respiratory pathogens. Often overlooked in animal experiments is the fact that the sex and hormonal status of an individual can regulate inflammatory responses and the development of immunopathology during *Nocardia* infection. In the present study, we sought to use sex-based and E2-manipulated mouse models of *N. farcinica* IFM 10152 infection to clarify the efficiency of E2 in inflammation and bacterial clearance. The underlying mechanisms by which E2 affects *Nocardia* infection were then initially elucidated at the cellular level. The ultimate goal of this study was to improve the understanding of the mechanism of sex differences in inflammatory lung diseases associated with *Nocardia* infection and provide evidence for optimizing clinical preventive measures and treatments for each sex.

## Materials and Methods

### Mice and Ethics Statement

Female and male C57BL/6 mice (6–8 weeks of age) were purchased from SPF Biotechnology Co., Ltd. (Beijing, China) and bred under specific pathogen-free conditions according to the guidelines. All procedures were approved by the Ethics Review Committee of the National Institute for Communicable Disease Control and Prevention at the Chinese Center for Disease Control and Prevention.

### Bacteria and Infection of Mice

*N. farcinica* IFM 10152 was purchased from the German Resource Centre for Biological Materials. Bacteria were grown in BHI broth (Oxoid Ltd, Hants, UK) at 37°C to exponential phase before experiments. Female and male C57BL/6 WT mice were injected intraperitoneally with a uniform bacterial suspension (100 µl) containing approximately 2×10^8^ colony-forming units (CFU), and mortality was assessed for 14 consecutive days. For inflammatory studies, 1×10^7^ CFU of *N. farcinica* IFM 10152 (50 µl) or 50 µl PBS was intranasally infected under anesthesia.

### Weight and Body Temperature

Mouse weight and body temperature were quantified immediately prior to *N. farcinica* IFM 10152 infection and 1 day post-infection. Mice were weighed to hundredth of a gram accuracy, and body temperature was monitored with an Animal Thermometer (KEW, Nanjing, China), which steadily assesses rectal temperature to the nearest 0.1°C in 3–5 seconds.

### Bronchoalveolar Lavage Fluid and Lung Homogenates Sample Collection

After the mice were sacrificed by cervical dislocation, pulmonary bronchoalveolar lavage fluid (BALF) was obtained through 3 successive lavages of the bronchi with 1 mL of ice-cold PBS under a sterile environment, and the protein content was assessed using Bradford reagent (TIANGEN, Beijing, China) following the manufacturer’s instructions. Whole lung and spleen tissue was collected and homogenized in 1 ml of PBS. For enumerating bacterial counts, serial dilutions of homogenate were plated on BHI agar plates, and the number of *N. farcinica* IFM 10152 CFUs was counted after 48 hours of incubation at 37°C.

### Lung Histopathology

Lungs were fixed in 4% paraformaldehyde overnight, embedded in paraffin and cut into 5-μm sections. Slides were stained using hematoxylin and eosin and then viewed using a biological microscope (Nikon, Eclipse Ci-L, Japan) according to the manufacturers’ instructions.

### Cytokine Measurements

For time course experiments, animals were randomly assigned to be euthanized at 1, 7, or 14 days. Supernatants from lung homogenates were used to measure IL-4, IL-6, IL-10, IL-12, TNF-α and IFN-γ by quantitative ELISA (BD OptEIA™, San Diego, CA, USA). The assays were conducted as recommended by the manufacturer, and all cultures were processed in triplicate.

### Growth Curve

To examine the direct effect of E2 on *N. farcinica* IFM 10152 growth, E2 at different concentrations (10 nM, 50 nM, 250 nM) was added to the growth curve plate at 37°C. The OD_600_ value was tested half an hour for 48 hours using an automatic growth curve analyzer (Bioscreen, Finland).

### Ovariectomy and Estrogen Treatment

Ovaries of 6-week-old female mice were removed through bilateral incisions over the dorsum under anesthesia. For the sham operation, the ovaries were identified, and an equal volume of paraovarian adipose tissue was removed. Ten days after incisions were sutured, mice were injected subcutaneously with 100 μL of sesame seed oil with or without 100 ng of E2 (Sigma, St. Louis, MO) at 10:00 am for ten consecutive days before *N. farcinica* IFM 10152 infection. Then, E2 concentrations in serum were measured at 1 day postinfection using a Mouse E2 ELISA kit (MEIMIAN, Wuhan, China). Bacterial burden and protein content were determined as described above.

### Cell Isolation and Culture

Primary alveolar macrophages were obtained by centrifuging BALF and were resuspended in phenol-free DMEM (BBI, Shanghai, China) supplemented with 10% fetal bovine serum (FBS; Gibco, USA). After 2 hours of incubation in the cell culture dishes at 37°C, the supernatant was discarded, and the adherent cells obtained were cultured with new medium. The mouse and human cell lines RAW264.7 and A549 (National Infrastructure of Cell Line Resource, Beijing, China) were cultured in phenol-free DMEM with 10% FBS at 37°C. In each experiment, wells were washed three times with PBS and seeded with or without 50 nM E2 at 37°C for 16–18 h in a CO_2_ incubator before infection, and the cell suspension containing *N. farcinica* IFM 10152 was treated at an MOI of 10:1.

### Adhesion and Invasion

For the adhesion assay, A549 and RAW264.7 cells were seeded into 24-well microplates with or without round glass coverslips at a density of 3×10^5^ cells per well. After 1 h postinfection at 37°C, for electron microscopic observation, cells were washed three times with PBS and then fixed with methanol for 8 min at room temperature. After methanol was removed, cells were stained with Giemsa stain solution and left for 30 min at room temperature. Coverslips were then washed and removed from the petri plate and examined using an Echo Revolve Generation 2 (ECHO, USA). For bacterial adhesive determination, cells were washed twice with PBS to eliminate extracellular bacteria and lysed with 1 ml of H_2_O to disrupt the cells and release the intracellular bacteria. Finally, cell lysates were serially diluted 10-fold for CFU determination and plated on BHI agar plates.

For the invasion assay, A549 and RAW264.7 cells were incubated in a 24-well microplate at a density of 3×10^5^ cells per well, and primary alveolar macrophages were incubated at a density of 3×10^4^ cells per well. After 1 h of infection, cells were washed twice with PBS to eliminate extracellular bacteria and incubated in DMEM containing 50 μg/ml amikacin (20) and 2% FBS. For bacterial survival determination, cell lysates were plated on BHI agar plates after serially diluted. After 48 h of incubation, the colonies were counted.

### Cytotoxicity Assay

Cytotoxicity assays of the E2-treated group and control group at 8 h postinfection were conducted using a CytoTox 96^®^ Non-Radioactive Cytotoxicity Assay (Promega, Madison, USA) as previously described (21). The absorbance data at 490 nm were collected using a microplate reader (BioTek, USA) according to the manufacturer’s instructions.

### Estrogen Receptor Antagonist

To block estrogen receptors, RAW264.7 cells were pretreated for 1 h at 37°C with the ER antagonist ICI 182780 (which blocks both nuclear and nonnuclear ERs, APEBixo, USA), the ERα-specific antagonist MPP (APEBixo, USA) or the ERβ-specific antagonist PHTPP (APEBixo, USA) prior to E2 exposure. CFU determination in RAW 264.7 cells was counted as described before.

### Western Blot Analysis

For Western blot analysis, whole-cell extracts were harvested using RIPA lysis buffer (strong) (CWBIO, Beijing, China) containing protease inhibitor cocktail (CWBIO, Beijing, China) and phosphatase inhibitor cocktail (CWBIO, Beijing, China) at 30 min, 60 min, and 120 min postinfection, separated by SDS–PAGE and transferred onto PVDF membranes (Millipore, Darmstadt, Germany). Subsequently, the membranes were incubated with primary antibodies against monoclonal mouse anti-β-actin (1:4000, TransGen, China), rabbit anti-p-p44/42 MAPK (1:1000, CST, USA), rabbit anti-p-SAPK/JNK (1:1000, CST, USA) or rabbit anti-p-p38 (1:1000, CST, USA) overnight, followed by incubation with HRP-conjugated goat anti-rabbit IgG (1:1000, Beyotime, China) or HRP-conjugated goat anti-mouse IgG (1:4000, ZSGB-BIO, China). Finally, the bands were visualized using Amersham^®^ Hyperfilm^®^ ECL™ and MP Autoradiography Films (GE Healthcare).

### MAPK Inhibitor

To block MAPK signaling, RAW264.7 cells were pretreated for 1 h at 37°C with inhibitors of 20 μM p38 (SB 203580, Sigma, USA), 20 μM ERK (PD 98059, Sigma, USA) or 20 μM JNK (SP 600125, Sigma, USA) prior to E2 exposure. CFU determination was counted as described before.

### Statistical Analysis

Survival and growth curves were analyzed using GraphPad Prism 9.0.0. Group means and standard deviations (SDs) were analyzed by Student’s *t* test. Grayscale values of protein bands were analyzed by Image J. For all tests, difference was considered statistically significant if the *p* value is less than 0.05.

## Results

### Female Mice Show Higher Mortality From *N. farcinica* Infection Than Male Mice

**Figure 1:**
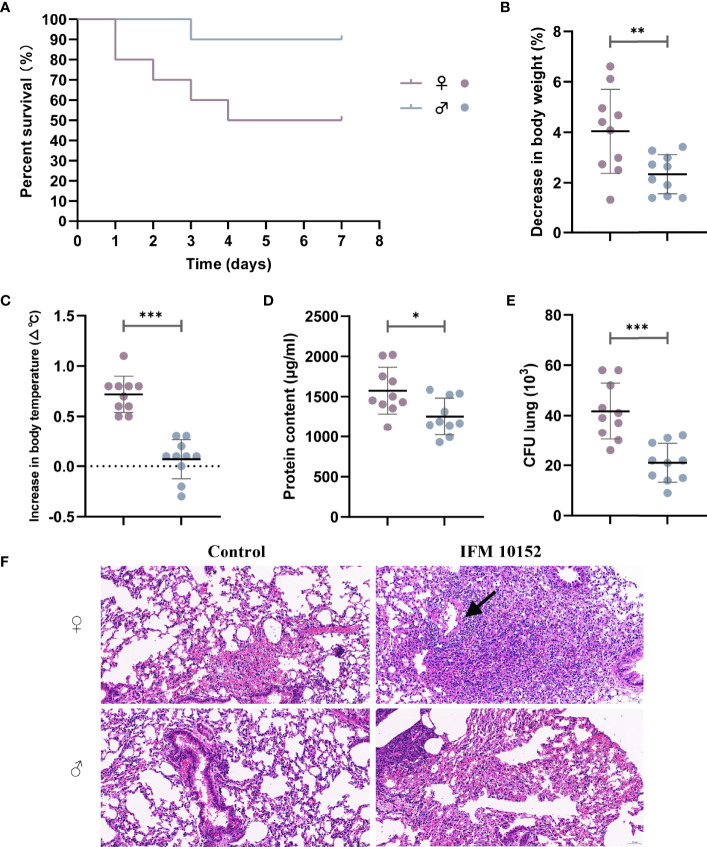
Female mice are more susceptible to *N. farcinica* infection. **(A)** Female mice (n = 10) and male mice (n = 10) were injected intraperitoneally with 2×10^8^ CFU (100 µl) of *N. farcinica* IFM 10152, and mortality was assessed for 14 days until no additional deaths were observed. **(B–F)** Female mice (n = 12) and male mice (n = 12) were infected intranasally with 1×10^7^ CFU (50 µl) for 24 hours, and control groups were infected with 50 µl of PBS. **(B)** Change in body weight of mice. **(C)** Change in body temperature. **(D)** Protein content in BALF. **(E)** Bacterial burden in lung homogenates. **(F)** Representative H&E-stained lung sections of mice. Scale bars: 100 µm. Each point represents a mouse. Lines display means with SEM. Data are from 3 independent experiments. *P < 0.05, **P < 0.01, and ***P < 0.001.

Following *N. farcinica* IFM10152 inoculation, female mice died significantly earlier than male mice, with survival differences noted as early as 24 h post-infection (**Figure 1A**). While 90% of male mice were able to survive, only 50% of female mice survived for 14 consecutive days.

### Female Mice Show Increased Disease Severity Upon *N. farcinica* Infection

To determine potential differences in lung infection and inflammation between sexes, we further established a nonlethal acute pneumonia model in age-matched C57BL/6 female and male mice with 1 ×10^7^ CFU *N. farcinica* IFM 10152 by intranasal infection. At 24 h postinfection, we observed that female mice had decreased body weight (4.04% decrease vs. 2.32%, **Figure 1B**) and increased body temperature (0.7°C increase vs. 0.1°C, **Figure 1C**) compared with male mice. Except for the dramatic physical changes, female mice had more abundant protein content than male mice in their airways (*P* < 0.05), as a sign of lung injury (**Figure 1D**). Consistent with the poor prognosis, female mice displayed a higher bacterial burden in lung tissue (*P* < 0.001) than male mice (**Figure 1E**), but no bacteria were found in the spleen tissue in either male or female mice (data not shown).

Examination of lung histopathology revealed exacerbated pathology in the *N. farcinica* IFM 10152-infected female mice compared with male mice (**Figure 1F**). The lungs of infected female mice showed marked thickening of the alveolar wall with large amounts of lymphocyte, neutrophil and macrophage infiltration, and some necrotic cell debris and hemorrhaging were also observed in the bronchial lumen. In addition, there was also evidence of inflammatory cells infiltrating into a ring around the vessel, forming a vascular sleeve (**Figure 1F**; black arrow). Male mouse-infected lungs showed evidence of lymphocyte and neutrophil infiltration, as well as slight thickening of the alveolar wall. And no obvious necrotic cell debris and vascular sleeves were observed in the bronchial lumen.

### Female Mice Display Higher Cytokine Production in Response to Respiratory *N. farcinica* IFM 10152 Infection

**Figure 2:**
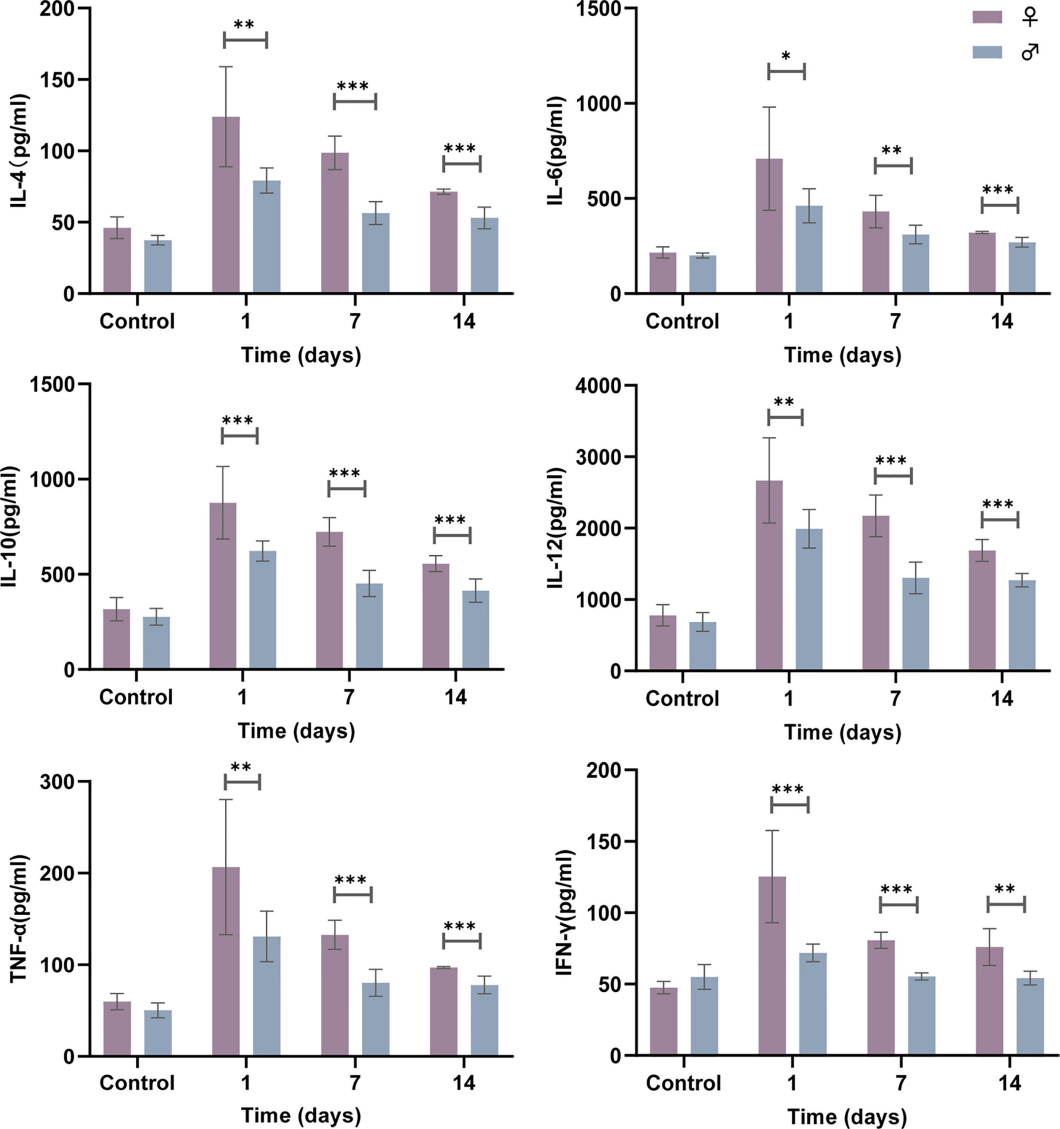
Female mice display higher cytokine production following *N. farcinica* IFM 10152 infection. Female mice (n = 32) and male mice (n = 32) were infected intranasally with 1×10^7^ CFU (50 µl) for 1, 7, and 14 days, or 50 µl PBS for 1 day, and cytokine levels (IL-4, IL-6, IL-10, IL-12, TNF-α and IFN-γ) in the lung supernatant were measured by ELISA. Lines display means with SEM. Data are from 2 independent experiments. *P < 0.05, **P < 0.01, and ***P < 0.001.

The production of cytokines by innate immune cells can also differ between the sexes in response to different stimuli, including bacterial infections. Analysis of the cytokine levels in the lung supernatant showed that at 1, 7 and 14 days postinfection, female mice had significantly elevated cytokine production levels (IL-4, IL-6, IL-10, IL-12, TNF-α and IFN-γ) in response to *N. farcinica* IFM 10152 compared with male mice, although all cytokines showed a decreasing trend during the days(**Figure 2**). These massive cytokine levels, which persisted for more than two weeks, are likely a consequence of the poor prognosis in the female mice.

### E2 Cannot Alter the Growth Curve of *N. farcinica* IFM 10152

**Figure 3:**
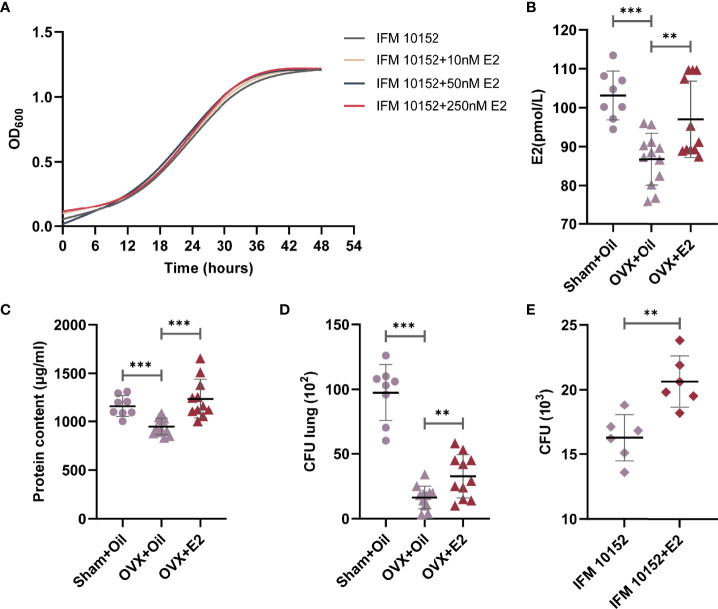
E2 supplementation increases ovariectomized female mouse susceptibility to *N. farcinica* IFM 10152 lung infection. **(A)** Growth curve of *N. farcinica* IFM 10152 in BHI with 10 nM, 50 nM, and 250 nM for 48 hours. **(B–D)** Female mice were treated with sham ovariectomy with sesame seed oil (n=8), ovariectomy with sesame seed oil (n = 12), or ovariectomy with E2 (n = 12) prior to challenge with *N. farcinica* IFM 10152. **(B)** E2 levels in serum. **(C)** Protein content in BALF. **(D)** Bacterial burden in lung homogenates. **(E)** Bacterial survival in alveolar macrophages treated with or without E2. Lines display means with SEM. Data are from 2 independent experiments. **P < 0.01, and ***P < 0.001.

Sex steroid hormones have been described to directly influence bacterial growth and metabolism. To test whether E2 could exert a direct effect on *N. farcinica* IFM 10152 growth, bacteria were grown in brain-heart infusion agar (BHI), with and without E2. Under the conditions tested, we found no quantitative change in the growth of *N. farcinica* IFM 10152, regardless of E2 concentration (**Figure 3A**).

### Treatment With E2 Increases Ovariectomized Female Mouse Susceptibility to *N. farcinica* IFM 10152 Lung Infection

Having identified no direct effect of E2 on *N. farcinica* IFM 10152 growth, we sought to determine whether E2 impacted the host inflammatory response. Female mice were sham ovariectomized (supplemented with sesame seed oil) or ovariectomized (supplemented with sesame seed oil or E2 at physiological doses) prior to challenge with *N. farcinica* IFM 10152. The results showed that serum E2 levels in female mice decreased significantly after ovariectomization but increased after exogenous E2 supplementation (**Figure 3B**). The E2 effect was confirmed by measuring the protein content in BALF and counting the bacterial burden in lung tissues. Concordant with the changes in E2 levels, E2-treated ovariectomized mice had a significant increase in protein content compared with oil-treated ovariectomized mice, which was essentially the same as that of oil-treated sham ovariectomized mice (**Figure 3C**). Furthermore, the lung bacterial burden of ovariectomized mice was significantly higher after supplementation with E2, although it was still significantly lower than that of sham ovariectomized mice (**Figure 3D**). These data demonstrated that ovariectomized mice supplemented with E2 exhibited impaired bacterial clearance compared to oil-treated ovariectomized mice.

### E2 Contributes to the Growth of *N. farcinica* IFM 10152 in Alveolar Macrophages

Previous studies established that *Nocardia* grew as a facultative intracellular parasite in cultured alveolar macrophages (21, 22). We next determined the direct effects of E2 on *N. farcinica* IFM 10152 growth in alveolar macrophages by seeding *N. farcinica* IFM 10152 into phenol-free DMEM containing FBS with 50 nM E2 for 6 hours. The results showed that E2-treated cells had more *N. farcinica* CFUs in plates than controls (**Figure 3E**).

### E2 Facilitates Adhesion and Invasion of *N. farcinica* IFM 10152 Into Host Cells Dependent on Nuclear Estrogen Receptors

**Figure 4:**
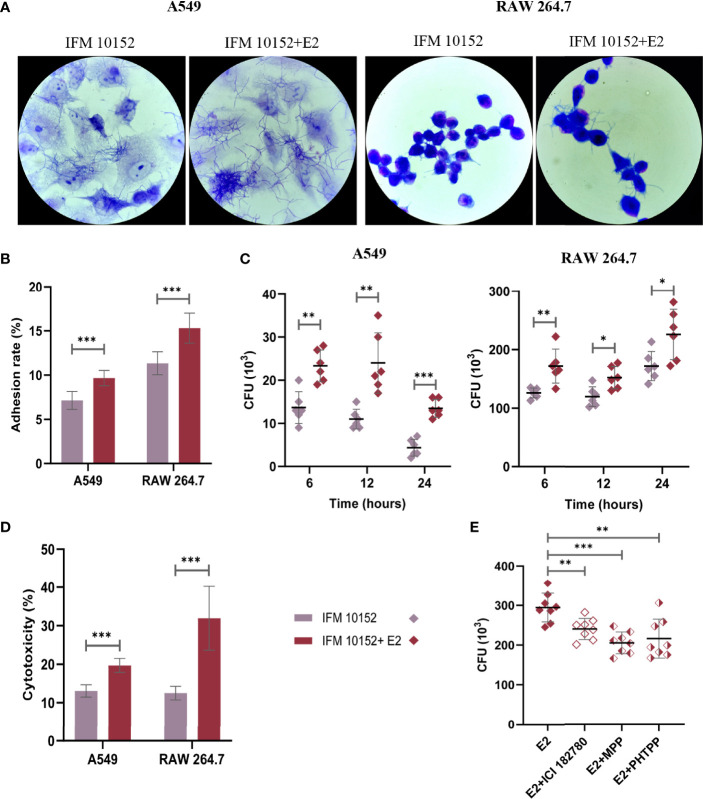
E2 facilitates adhesion and invasion of *N. farcinica* IFM 10152 into host cells through ERα and ERβ signaling. **(A–E)** A549 and RAW 264.7 cells were treated with or without 50 nM E2 for 16–18 h prior to *Nocardia* infection. Electron microscopic observation **(A)** and adhesion rate **(B)** of bacterial strains to A549 (left) and RAW 264.7 (right) cells after 1 h of infection. **(C)** Invasion of bacterial strains into A549 (left) and RAW 264.7 (right) cells after 6, 12, and 24 h of infection. **(D)** The cytotoxicity of *N. farcinica* IFM 10152 to A549 and RAW 264.7 cells after 8 h of infection. **(E)** Bacterial survival in RAW 264.7 cells treated with ICI 182780, MPP or PHTPP. Lines display means with SEM. Data are from 3 independent experiments. *P < 0.05, **P < 0.01, and ***P < 0.001.

To further evaluate the effect of E2 on *N. farcinica* IFM 10152 growth in cells, A549 and RAW 264.7 cells were imaged by electron microscopy after 1 h of infection. The results showed that *N. farcinica* IFM 10152 adhered and proliferated better in the E2-treated group than in the control group (**Figures 4A, B**). As such, the bacterial burden of *N. farcinica* IFM 10152 in the E2-treated groups was higher than that in the control group at 6, 12 and 24 h postinfection (**Figure 4C**). In addition, we observed that the cytotoxicity of *N. farcinica* IFM 10152 was significantly higher in the E2-treated group than in the control group in both A549 and RAW264.7 cells (**Figure 4D**). To determine whether the difference in cells treated with E2 versus vehicle was ER-specific, RAW 264.7 cells were seeded and supplemented with three ER antagonists. The increased bacterial burden in E2-supplemented RAW 264.7 cells was attenuated in the presence of ICI 182,780, MPP and PHTPP (**Figure 4E**). Taken together, these findings support a specific role for nuclear ERs in the impact of E2 on promoting *N. farcinica* IFM 10152 infection.

### E2 Promotes Bacterial Survival by Downregulating the Phosphorylation Level of the MAPK Pathway

**Figure 5:**
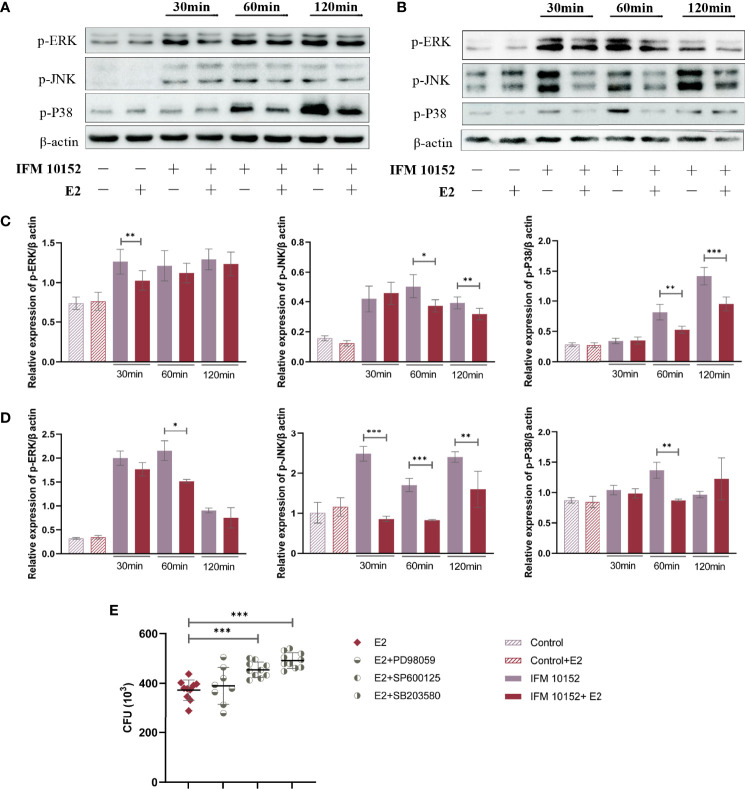
E2 promotes bacterial survival by downregulating the MAPK signaling pathway. A549 **(A)** and RAW 264.7 **(B)** cells were treated with or without 50 nM E2 for 16–18 h prior to *Nocardia* infection. Western blot analysis of the phosphorylation levels of ERK (p-ERK), JNK (p-JNK), and p38 (p-p38) after 30, 60, and 120 minutes of infection. The relative expression of each protein in A549 **(C)** and RAW 264.7 **(D)** cells was analyzed by Image J. **(E)** Bacterial survival in RAW 264.7 cells treated with 20 μM PD 98059, 20 μM SP 600125, or 20 μM SB 203580. Lines display means with SEM. Data are from 3 independent experiments. *P < 0.05, **P < 0.01, and ***P < 0.001.

To elucidate the mechanisms by which E2 promotes *Nocardia* survival in host cells, we examined mitogen-activated protein kinase (MAPK) activation in response to *N. farcinica* IFM 10152 infection. The results showed that the E2-treated group downregulated the phosphorylation levels of ERK (p-ERK), JNK (p-JNK), and p38 (p-p38) compared to the control group in both A549 and RAW 264.7 cells (**Figures 5A–D**). Then, we detected the relationship between bacterial survival in cells and the MAPK signaling pathway with MAPK inhibitors. The results showed increased bacterial survival in the SB 203580- and SP 600125-treated groups, although there was no detectable difference between the PD 98059-treated and control groups (**Figure 5E**). These results indicate that E2 promotes bacterial survival by inhibiting activation of the MAPK-mediated inflammatory response.

## Discussion

Sex differences in immunity to respiratory pathogens are evident in humans and experimental rodent models (23). The roles of sex differences and sex hormones have been investigated in experimental models of infection and inflammation with varied results. For many inflammatory-mediated pulmonary diseases, including *Mycobacterium tuberculosis* (24) and *Streptococcus pneumoniae* (25), male mice are more susceptible than female mice. However, female mice are more likely to be hospitalized and/or die following infection with other respiratory pathogens, including *Pseudomonas aeruginosa* (26, 27) and *Acinetobacter baumannii* (28).

Given the current importance now placed on utilizing both males and females in research, we designed an innate immune response model of *N. farcinica* IFM 1015*2* respiratory tract infection in male and female mice. The results showed significantly higher mortality in female mice infected with a lethal dose of *N. farcinica*. Similarly, infection with a nonlethal dose resulted in worse outcomes in female mice than in male mice. The data from the present study illustrate that female mice displayed unstable physical changes, severe lung damage, elevated inflammatory cytokine responses and overall lower bacterial clearance in the lungs after 24 h of infection. In addition, we observed that cytokines in the lung supernatant of female mice were higher than those of male mice for 2 weeks after infection. These massive cytokine levels increase body temperature and excessive inflammatory response, which may be associated with poor prognosis in female mice. These data support and extend the hypothesis that although there is a higher prevalence of *Nocardia* infection in males, females tend to suffer a poor outcome.

Previous studies have focused mostly on sex differences in the incidence of *Nocardia* infection, but little attention has been given to sex differences in prognosis. We observed higher mortality for females than males in some well-documented reports. Rafiei N et al. (29) studied 10 males and 10 females with *Nocardia* infection in Queensland from 1997 to 2015. After years of follow-up, it was found that the death toll of females was six, which was higher than that of males (two). Sex differences in disease outcome are likely mediated by multiple factors, including sex hormones, glucocorticoids and sex chromosomal genes (30). E2 has been shown to have both proinflammatory and anti-inflammatory roles in host resistance to pathogen infections (18, 19). In the present study, ovariectomized female mice shared a lower bacterial burden in the lungs than sham ovariectomized female mice, and exogenous administration of E2 increased the bacterial burden in ovariectomized female mice, which indicates that E2 can directly or indirectly impair the ability of *Nocardia* clearance in mice. Moreover, although the E2 level in E2-treated ovariectomized female mice essentially reached normal levels, the bacterial burden in the lungs was still significantly lower than that in sham ovariectomized female mice, which indicates the difficulty in reproducing natural E2 function *via* manipulation *in vivo*. Similarly, we observed that E2 can bind nuclear ER-α and ER-β to promote the invasion of *Nocardia* into host cells, resulting in severe cellular damage. However, a previous study showed that E2-treated mice can effectively inhibit the growth of bacterial grains after plantar pad infection with *N. brasiliensis*, demonstrating the protective effect of E2 in mice (16). These different results could be due to the differences in the experimental subjects and experimental approaches, such as the *Nocardia* strains used and/or stimuli employed.

In most respiratory diseases, in general, the severity of symptoms was related to the innate immune response triggered during the early period of infection (31). MAPKs are key factors mediating cellular activities such as cell differentiation, stress responses, apoptosis, and immune defenses to many external stimuli (32). Our observations indicate that E2 can significantly downregulate the phosphorylation level of the MAPK pathway. Further research also showed that downregulation of the MAPK signaling pathway was conducive to bacterial survival in host cells. These data provide evidence that downregulation of the MAPK signaling pathway is one of the mechanisms by which E2 promotes the survival of *N. farcinica*.

In the present study, we demonstrated that the differential susceptibility to *Nocardia*–induced pneumonia between sexes is partly based on E2. Our data provide evidence for determining the specific therapeutic target for sex hormone manipulation. Sex-based differences need to be taken into account in subsequent research and in the understanding of nocardiosis. Ongoing work in our laboratory is further elucidating the difference in antibody production between male and female mice following *Nocardia* infection and then delineating the underlying mechanism from the perspective of humoral immunity.

## Conclusion

Despite the higher prevalence of *Nocardia* infection in males, females tend to suffer a poor outcome with increased mortality, elevated lung bacterial loads and an exaggerated pulmonary inflammatory response. 17β-Estradiol can promote bacterial survival by downregulating the host MAPK signaling pathway, which is one of the mechanisms responsible for this sex difference.

## Data Availability Statement

The original contributions presented in the study are included in the article/supplementary material. Further inquiries can be directed to the corresponding author.

## Ethics Statement

The animal study was reviewed and approved by Ethics Review Committee of the National Institute for Communicable Disease Control and Prevention at the Chinese Center for Disease Control and Prevention.

## Author Contributions

LH: Conceptualization, Investigation, Writing - original draft. XJ: Methodology, Writing - review & editing. XL: Methodology, Writing - review & editing. SX: Investigation, Data curation. FL: Investigation, Data curation. YC: Software, Resources. XQ: Software, Resources. LS: Formal analysis, Validation. ZL: Supervision, Writing - review & editing, funding acquisition. All authors contributed to the article and approved the submitted version.

## Funding

This work was supported by National Key Research and Development Program of China (grant number: 2019YFC1200601 and 2019YFC1200700), and National Natural Science Foundation of China (grant number: 82073624).

## Conflict of Interest

The authors declare that the research was conducted in the absence of any commercial or financial relationships that could be construed as a potential conflict of interest.

## Publisher’s Note

All claims expressed in this article are solely those of the authors and do not necessarily represent those of their affiliated organizations, or those of the publisher, the editors and the reviewers. Any product that may be evaluated in this article, or claim that may be made by its manufacturer, is not guaranteed or endorsed by the publisher.
